# A Case of an Acquired Factor VIII Inhibitor Complicated by Multiple Treatment-Related Opportunistic Infections and Review of the Literature

**DOI:** 10.1155/2013/703027

**Published:** 2013-12-18

**Authors:** Anna L. Hutchinson, Yi Ling Tan, Giselle Kidson-Gerber

**Affiliations:** ^1^Prince of Wales Hospital, Barker Street, Randwick, NSW 2031, Australia; ^2^Department of Medicine, University of New South Wales, Botany Street, Sydney, NSW 2052, Australia

## Abstract

This case report describes a patient with an idiopathic acquired Factor VIII inhibitor and severe bleeding. She was treated with rituximab after failing first-line treatment with steroids and cyclophosphamide. Two months following rituximab treatment, our patient developed a succession of severe opportunistic infections requiring intensive care unit admission. Over a period of 12 weeks she required treatment for *Pseudomonas aeruginosa* septicaemia, herpes simplex gingivostomatitis and pharyngotonsillitis, clostridium difficile-related diarrhoea, systemic cytomegalovirus infection, pneumocystis jiroveci, and invasive pulmonary aspergillosis lung infections. After significant rehabilitation, the patient was finally discharged following a 5-month admission. This case highlights the complexity of balancing a life-threatening condition with the side effects of treatment. It also raises the issue of routine prophylaxis for immunosuppression in nonmalignant conditions, which will become a common dilemma with the expanding indications for rituximab use.

## 1. Introduction

The incidence of acquired inhibitors against FVIII is 1 to 4 per million/year in the nonhemophilic population [1]. In affected patients, the rate of severe bleeds is up to 90%, with mortality rates between 8 and 22% [2]. This condition is generally diagnosed after detection of an isolated prolonged activated partial thromboplastin time (APTT), with failure to correct on mixing studies, and subsequent identification of reduced FVIII levels and presence of FVIII inhibitor. Most cases of acquired FVIII inhibitor are idiopathic, but up to 50% are associated with autoimmune diseases, malignancies, medications, or the postpartum period [2, 3].

Treatment of acute bleeding episodes is tailored according to inhibitor titre, site, and severity of bleeding. In high-titre patients, bypassing agents such as recombinant factor VIIa or FVIII inhibitor bypass activity (FEIBA) are indicated [1]. In patients with a low titre inhibitor (i.e., <5 Bethesda units or BU), plasma-derived or recombinant human FVIII can be used [1].

Current first-line treatment for eradication of FVIII inhibitor is oral corticosteroid [3, 4]; this may be combined with cyclophosphamide [3]. Although combination with cyclophosphamide results in a greater remission rate than steroid alone, the increased rate of neutropenia-related infection means that the overall mortality rate is unchanged [4]. There is increasing evidence for the efficacy of rituximab (RTX) in those who fail first-line treatment or as first-line treatment for patients in whom corticosteroids and chemotherapeutic agents are contraindicated [3, 5–7]. RTX is a chimeric anti-CD20 monoclonal antibody widely used in the treatment of autoimmune disorders. It leads to the depletion of CD20+ B cells, which is hypothesised to interrupt autoantibody production. Berezné et al. report that RTX can be considered as first- or second-line treatment, either alone or in combination with cyclophosphamide [7]. Treatment of refractory FVIII inhibitor may also include intravenous immunoglobulin administration and immunoadsorption, particularly when bleeding cannot be controlled [4, 8].

## 2. Case Study 

A 66-year-old woman with a background of type 2 diabetes mellitus was referred to the hematology service with bleeding after investigatory colonoscopy for symptomatic anemia. After colonoscopy she developed melena, hematuria, extensive subcutaneous hemorrhage, and a subsequent retroperitoneal hematoma. The severity of her bleeding required more than 30 packed red cell transfusions during her admission, FVIII concentrate, and tranexamic acid. There was no personal or family history of bruising or bleeding, and no underlying malignancy or autoimmune disorders were detected. HIV testing was negative.

The APTT was 79 seconds (reference range 25–37 seconds) with previously normal APTTs. Specific investigations demonstrated a strong FVIII inhibitor (234 BU) and residual FVIII activity of <1% (reference range: 50–150%). Initial management involved high-dose oral prednisone 50 mg daily and cyclophosphamide 100 mg daily. The prednisone was continued for 3 months and weaned to cessation over the fourth month. The cyclophosphamide was continued for 3 months and then ceased. No routine antimicrobial prophylaxis was concurrently given.

Three weeks following treatment initiation there was no improvement in APTT, FVIII inhibitor levels, or FVIII levels. Four cycles of RTX 375 mg/m^2^ weekly were initiated. Six weeks after commencement of RTX treatment, there was improvement of the APTT, FVIII inhibitor level, and FVIII levels (see Table 1). At 5 months after RTX treatment, the APTT and FVIII levels had normalised.

Two months after RTX treatment commenced and whilst receiving ongoing cyclophosphamide and prednisone, the patient required resuscitation for *Pseudomonas aeruginosa* septicemia. This was followed within a week by herpes simplex gingivostomatitis and pharyngotonsillitis and diarrhoea secondary to clostridium difficile infection. Pancytopenia developed with a neutrophil nadir of 0.9 × 10^9^/L.

Two weeks later, pneumocystis jiroveci and invasive pulmonary aspergillosis were diagnosed on bronchoscopy washings and brushings. Clinical cytomegalovirus (CMV) infection was confirmed by positive CMV nucleic acid testing of lung biopsy and serum and urine PCR. The prednisone dose was weaned and ceased. Cyclophosphamide was also ceased. Multiple intensive care unit admissions with respiratory support were necessary to manage these complications, and multiple courses of antibiotics and antifungals were required. After a five-month admission requiring significant rehabilitation, the patient was discharged. The factor VIII inhibitor remains in remission.

At presentation to hospital the patient was found to have hypogammaglobulinemia (IgG level of 4.75 mg/dL, normal range 7–16 mg/dL). She received 3 doses of intravenous immunoglobulin 4 weeks apart. Interestingly, she was hypogammaglobulinemic prior to RTX and remains hypogammaglobulinemic 3 years after this episode. The cause of this is unknown; her IgA, IgG, and IgM are all below the reference range.

The patient was taking metformin 1 gram twice daily and gliclazide 160 mg twice daily at the time of admission. Her blood sugar level (BSL) control was poor prior to treatment with prednisone (range 7–20 mmol/L) and deteriorated further with steroids (range 13–24 mmol/L). Despite additional sliding-scale short- and intermediate-acting insulin, her BSL control remained unsatisfactory throughout her admission (range 4–20 mmol/L).

## 3. Discussion

This case highlights the complexity of balancing a potentially life-threatening condition with the side effects of therapy. Disease control was only achieved after administering second-line therapy, however at the cost of more infections. Severe immunosuppression is an accepted risk in the management of malignancy and organ transplantation, but less so with autoimmune conditions. Our patient's immunosuppression led to successive severe infections: over a period of 12 weeks she suffered from clinically significant infections with *Pseudomonas aeruginosa* septicaemia, herpes simplex, clostridium difficile, pneumocystis jiroveci, invasive pulmonary aspergillosis, and systemic CMV.

Published case reports document the efficacy of RTX in the treatment of refractory FVIII inhibitors [1, 9, 10]. Wiestner et al. treated 4 consecutive patients with acquired FVIII inhibitor with RTX: 3 in combination with corticosteroids and one with RTX as monotherapy. In all cases, the inhibitor resolved within several weeks and the patients remained in remission during followup of 7 to 12 months [9]. The majority of current literature involves using RTX as second-line treatment and as combination rather than monotherapy [1, 5, 11]. RTX may be adequate to effectively treat low inhibitor level patients, but those with high level inhibitors (>100 BU/mL) generally require the addition of cyclophosphamide and corticosteroids [11].

Since the advent of such agents as FEIBA, recombinant factor VIIa, and desmopressin, the complications of treatment for acquired FVIII inhibitor have become a greater cause of morbidity and mortality than the risk of bleeding itself [8, 11]. Case reports in the literature document the side-effects of treatment; however, none describe complications as severe as outlined in this case. We support the efficacy of RTX; however, we wish to caution against the possible complications. We propose that the addition of RTX to an established immunosuppressant regime of prednisone and cyclophosphamide played a significant role in the development of successive life-threatening and opportunistic infections, whilst acknowledging contribution by poorly-controlled diabetes and hypogammaglobulinemia. A causal relationship cannot be confirmed, however, due to the administration of additional immunosuppressive therapy.

Several case reports regarding the use of RTX have highlighted its potential to cause cytopenias, particularly when given in combination with other chemotherapeutics [12]. In 72 patients with non-Hodgkin's lymphoma treated with RTX, 30% developed neutropenia [12]. Of these 21 patients, 19% suffered a major infection. These infections included *P. carinii* pneumonia, CMV reactivation, mycobacterial pneumonia, CMV pneumonitis, and bacterial pneumonia [12]. Neutropenia typically develops greater than 4 weeks following RTX treatment, in keeping with our patient's presentation [13], and resolves after an average of 11 weeks [12].

There have been multiple reports documenting the potential for the development of opportunistic infections in RTX-treated patients, in particular viral infections. In a case series of 64 patients with severe viral infections following combination RTX and chemotherapy, the range of viruses encountered included hepatitis B (HBV, *n* = 25), CMV infection (*n* = 15), varicella zoster virus (VZV, *n* = 6), and others including herpes simplex virus (HSV) [14]. One-third of non-HBV infections were fatal [14].

A study of 77 patients who received RTX after renal-transplant found that 45% developed infections: bacterial, viral, and fungal infections were seen in 36%, 18%, and 17% of the cohort, respectively [15]. A control group of 902 patients who did not receive RTX had an infection rate similar to that of the RTX group; however, the mortality rate from infection was significantly lower in this group (1.6%) compared with that in the RTX group (9.1%). Infections occurred an average of 3 months after RTX treatment commenced and the most common were septic shock, *Escherichia coli* septicemia, pneumonia, pyelonephritis, CMV, HSV, HBV reactivation, candidemia, aspergillosis, pneumocystosis, and cryptococcal meningitis [15]. A review of RTX use in autoimmune diseases (excluding rheumatoid arthritis) across 25 studies involving 389 patients shows the incidence of serious infections to range from 3 to 33% [16]. The mortality rate from infectious complications was 9% overall [16].

RTX is known to cause neutropenia [12, 17], hypogammaglobulinemia [18], and thrombocytopenia [12] and to increase the risk of infection [18]. Neutropenia usually occurs greater than 4 weeks after treatment commencement; the median time to development is 10 weeks (range 3–23) and average duration 11 weeks (range 1–23+) [12]. Late onset neutropenia (LON) has been seen in RTX monotherapy but is more common when combined with chemotherapy; LON occurs in 7–30% of RTX-treated patients [12, 13, 17]. The pathogenesis of LON remains uncertain; however, bone marrow biopsy in these patients reveals maturation arrest [17]. The development of neutropenia is strongly associated with the number of RTX doses [12].

Hypogammaglobulinemia occurs with increasing frequency the more cycles of RTX are given and is seen in patients treated for autoimmunity as well as for malignancy [19]. Of interest, the different subclasses are affected to different extents. In a study of over 1000 patients, IgM levels fell by 10% after a single course, IgA by 4%, and IgG by 2% [19]. A review of the literature did not locate any articles linking poorly controlled diabetes or hypogammaglobulinemia to the list of infections suffered by this patient.

## 4. Teaching Points

We felt it was important to document this case to alert clinicians to the potential adverse effects of immunosuppression for nonmalignant conditions. RTX-treated patients should be closely monitored and promptly treated for opportunistic infections. Clinicians should be especially alert in patients with additional immune dysregulation and some patients may benefit from routine prophylaxis against viruses, bacteria, and fungi. We did not give antimicrobial prophylaxis to this patient; however, in retrospect it would have been appropriate. The combination of RTX with other immunosuppressive treatment rather than chemotherapy may have different ramifications, and this will become apparent with the expanding indications for RTX use.

## Figures and Tables

**Table 1:** APTT, FVIII level, and inhibitor level over time.

Time point	APTT (sec) [RR 25–37 sec]	FVIII inhibitor level (BU) [normal: 0 BU]	FVIII level (%) [RR 50–150%]
Presentation	83	234	<1%
3 weeks	77	509	<1%
9 weeks	36	118	22%
5 months	26.7	Not tested	187%

RR: reference range.
